# Supplementary figures and images for: Combining single-cell sequencing and spatial transcriptome sequencing to identify exosome-related features of glioblastoma and constructing a prognostic model to identify BARD1 as a potential therapeutic target for GBM patients
Source: Front Immunol. 2023 Aug 31;14:1263329. doi: 10.3389/fimmu.2023.1263329 (PMC10505933; doi:10.3389/fimmu.2023.1263329)

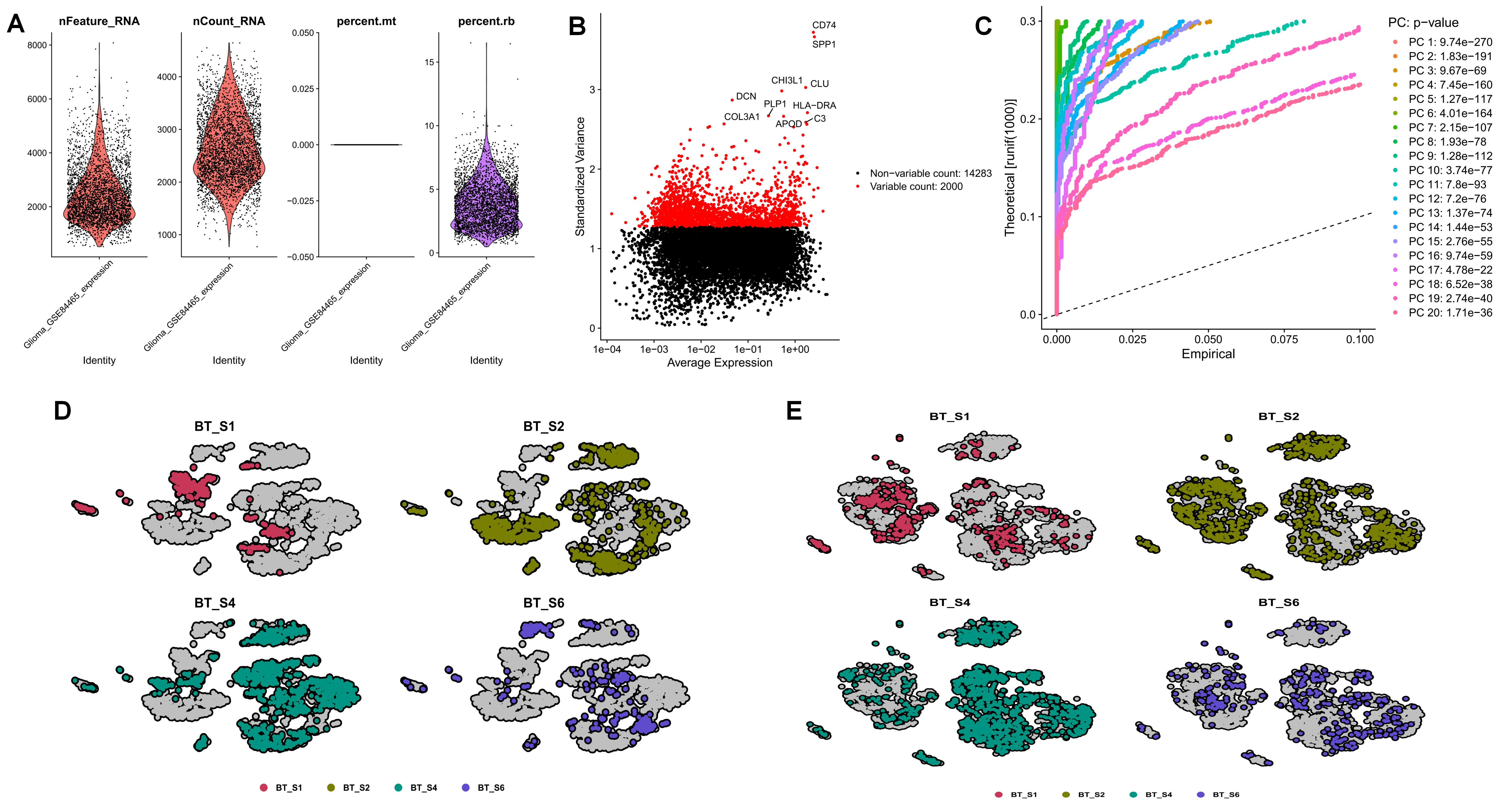

Supplement: Supplementary Figure 1 — quality control and principal component analysis of scRNA-seq. (A) Quality control of scRNA-seq data from GBM samples. (B) Variance plot showing 14,263 genes in all cells, with red dots representing the top 2,000 highly variable genes. (C) Principal component analysis was used to downscale and select the top 20 PCs.(D) t-SNE plots of four samples before over harmony to remove batch effects. (E) t-SNE plots of four samples after performing harmony to remove batch effects. [file Image_1.tif]

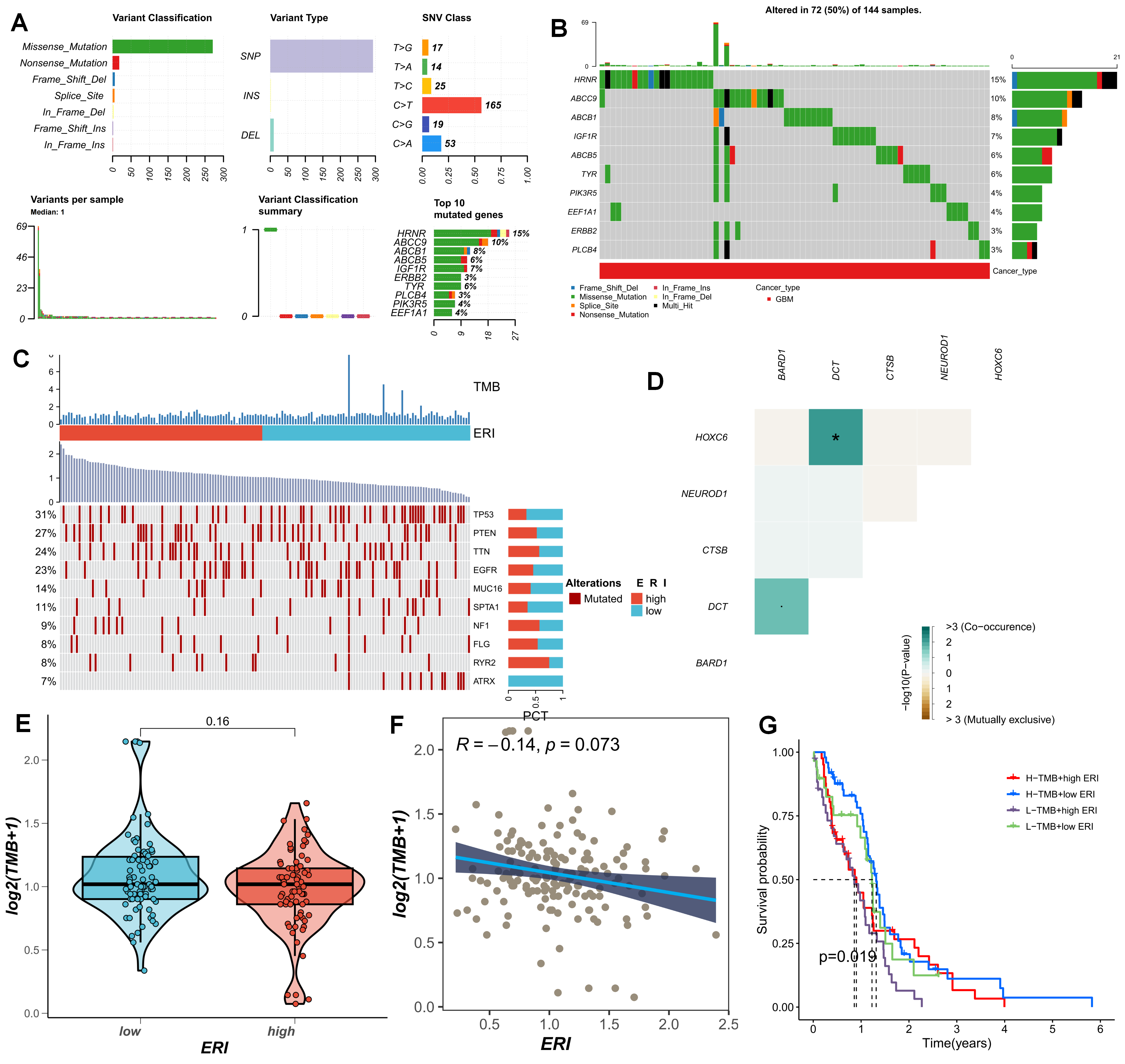

Supplement: Supplementary Figure 2 — Mutation landscape of GBM samples. (A) Description of the details of mutations in all 121 exosome-related genes, where the most common mutation type is a missense mutation. SNPs account for an absolute proportion of mutations compared to insertions or deletions, and C>A occurs more frequently than other types. The horizontal histogram lists the top 10 mutated genes in GBM. (B) Mutation landscape of all 121 exosome-related genes. (C) Mutation landscape of the top 10 genes in mutation frequency in the two ERI subgroups. (D) Co-mutation or co-exclusion relationships between model genes. (E) Comparison of tumor mutational load (TMB) between different subgroups. (F) Correlation analysis of ERI and TMB. (G) Survival differences among the four subgroups of H-TMB+ high ERI, H-TMB+ low ERI, L-TMB+ high ERI, and L-TMB+ low ERI. [file Image_2.tif]

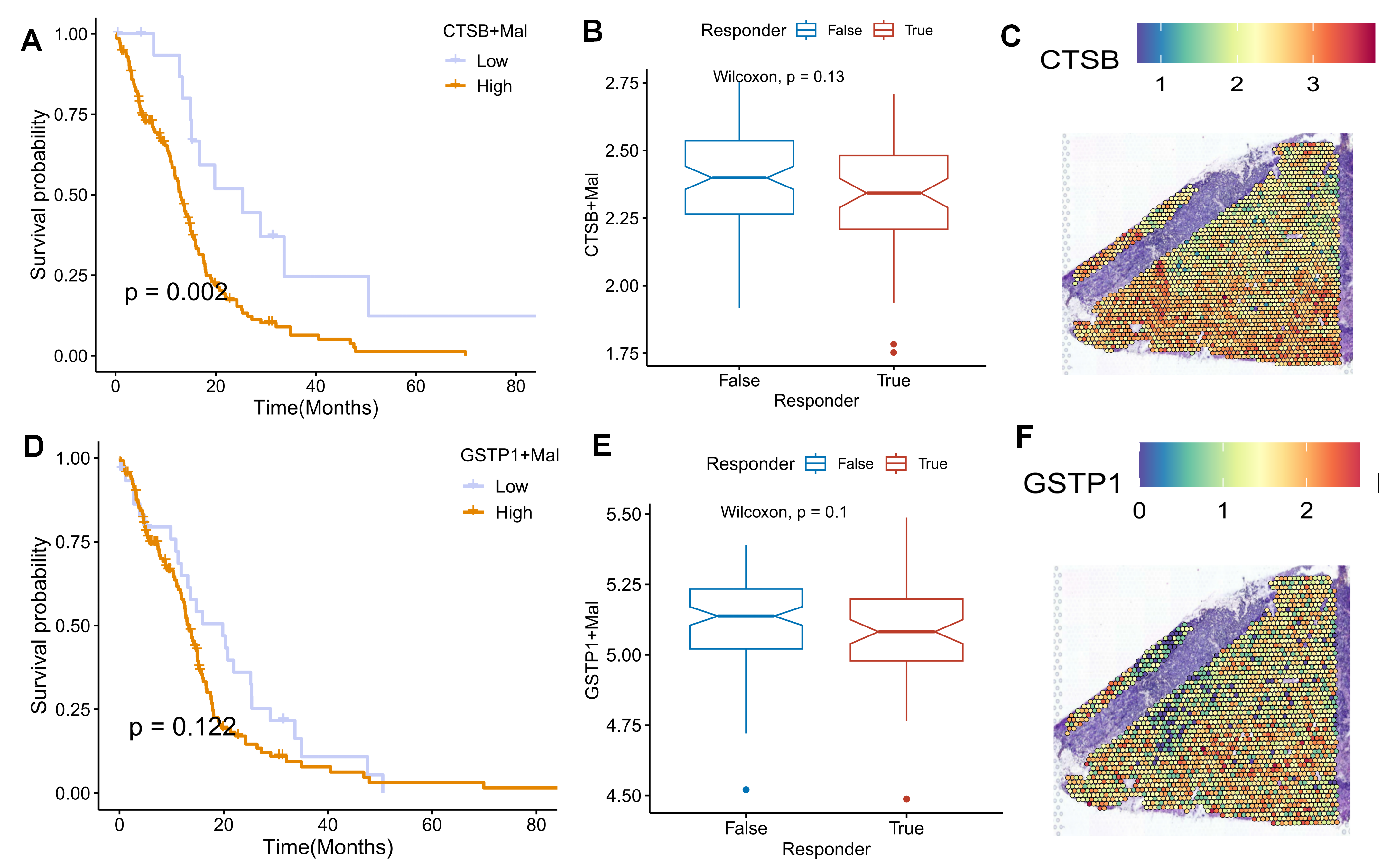

Supplement: Supplementary Figure 3 — Analysis of CTSB and GSTP1. (A) Kaplan-Meier survival curves for OS in patients in the high and low CTSB+ malignant cell expression group. (B) Prediction of response to immunotherapy based on the proportion of CTSB+ malignant cells. (C) Spatial map of CTSB expression. (D) Kaplan-Meier survival curves for OS of patients in the high and low GSTP1+ malignant cell expression group. (E) Prediction of response to immunotherapy based on the proportion of GSTP1+ malignant cells. (F) Spatial plot of GSTP1 expression. [file Image_3.tif]
